# AKL1, a botanical mixture for the treatment of asthma: a randomised, double-blind, placebo-controlled, cross-over study

**DOI:** 10.1186/1471-2466-7-4

**Published:** 2007-03-20

**Authors:** Michael Thomas, Jane Sheran, Natalie Smith, Sofia Fonseca, Amanda J Lee

**Affiliations:** 1 Department of General Practice and Primary Care, University of Aberdeen, Foresterhill Health Centre, Westburn Road, Aberdeen, AB25 2AY, UK; 2 Health Services Research Unit, University of Aberdeen, Health Sciences Building, Foresterhill, Aberdeen, AB25 2ZD, UK

## Abstract

**Background:**

Despite effective treatments, asthma outcomes remain suboptimal. Interest exists in complementary therapies, particularly in herbal remedies for asthma treatment, currently with inconclusive evidence of efficacy. The encapsulated botanical mixture AKL1 has anecdotal evidence of effectiveness in asthma.

**Methods:**

We performed a randomised controlled cross over study comparing the effectiveness of AKL1 with indistinguishable placebo as add-on therapy in patients uncontrolled on standard asthma treatment. Thirty two adult asthmatics completed a 36 week trial consisting of a 4 week single blind run in period, during which placebo was added to usual treatment, a 12 week double blind active phase in which subjects received AKL1 or placebo, a single blind 8 week washout period receiving placebo and a final 12 week double blind cross-over active treatment phase. Daily diaries were kept of peak expiratory flow and symptoms, and spirometry, validated symptom and health status questionnaire scores and adverse events were monitored at study visits. Paired T tests were used to compare the effects of placebo and AKL1 on outcomes. Changes in outcome measures over treatment phases are presented as means and 95% confidence intervals (CI) of means.

**Results:**

No significant differences in lung function (active-placebo) were found (Forced Expiratory Volume in 1 second: mean difference [95% CI] = 0.01 [-0.12 to 0.14] L, p = 0.9. Peak Expiratory Flow: -4.08 [-35.03 to 26.89]. L/min, p = 0.8).

Trends to clinical improvements favouring active treatment were however consistently seen in the patient-centered outcomes: Asthma Control Questionnaire mean difference (active – placebo) [95% CI] = -0.35 [-0.78 to 0.07], p = 0.10, Asthma Quality of Life Questionnaire mean difference 0.42 [-0.08 to 0.93], p = 0.09, Leicester Cough Questionnaire mean difference 0.49, [-0.18 to 1.16], p = 0.15.

Nine exacerbations occurred during placebo treatment and five whilst on AKL1. No significant adverse events were noted.

**Conclusion:**

AKL1 treatment was well tolerated. No significant improvements in lung function, symptoms, or quality of life were seen, although consistent trends were seen to improvements in patient-centered outcomes. Further studies are needed.

## Background

Asthma is a common chronic illness, and in spite of effective treatments outcomes remain sub-optimal [1]. Many patients harbour misgivings about conventional medical treatments for asthma, particularly inhaled corticosteroid (ICS) treatment [2,3], and adherence is frequently poor [4-6]. There is considerable public interest in complementary and alternative (CAM) treatments [7], including those for asthma, with surveys indicating a high level of use of all CAM treatments [8,9] but in particular of herbal treatments [10,11] for asthma. There are reports that 11% [12] to 40% [13] of people with asthma may use herbal remedies, with non-disclosure to orthodox practitioners being common.

The evidence of clinical effectiveness of herbal treatments for asthma is inconclusive; a systematic review [10] of 17 randomised controlled trials of herbal products for the treatment of asthma in 2000 found some promising results, but methodological deficiencies in most of these studies meant that uncertainly remains. Improvements in lung function and symptoms have been reported in some studies with different herbal agents [10]. More recent studies have also reported similar improvements in asthma outcomes [14-16], but the evidence for effective herbal treatments for asthma is not yet strong enough to make positive recommendations about specific herbal preparations or indeed about herbal treatment in general.

AKL1 is a novel agent containing a combination of botanical products developed as a treatment for asthma and formulated as a capsule. The botanical product contains a synthetically-derived phytochemical component of *Picrorrhiza kurroa*, apocynin, together with *Picrorrhiza kurroa, Zingiber officinale *and a standardized extract of *Ginkgo biloba*. The plant materials have been standardized against a known phytochemical marker, and additionally each ingredient was cross evaluated by the Medicinal Chemistry Department at the University of Utrecht to guarantee to standardization content as: Ginkgo biloba (standardized to contain ginkgo flavone glycosides – minimum 24%), Zingiber officinale (standardized to contain gingerols – minimum 5%), Picrorrhiza kurroa (enriched to contain apocynin – minimum 30%). Each 500 mg AKL capsule, which is a patented formulation, contains: Picrorrhiza kurroa (enriched to contain apocynin – min 28%) – 270 mg Ginkgo biloba (standardised to contain ginkgo flavone glycosides – min 24%) – 130 mg Zingiber officinale (standardised to contain gingerols – min 5%) – 100 mg. The treatment regime is 2 capsules twice a day. The various components of this mixture have been marked separately as health supplements, and the mixture was briefly sold as an over the counter health supplement prior to withdrawal pending full clinical evaluation. The mixture was formulated after extensive in-vitro and pragmatic clinical experimentation.

Anecdotal clinical evidence suggests that the botanical product has significant anti-asthma activity with patients reporting reduced frequency of attacks, reduced use of bronchodilators, reduced usage of inhaled corticosteroids and reduced hospitalization rates. Patients also report substantial reduction in cough and sputum production.

This study investigated the efficacy of this agent as 'add-on' therapy for adult patients whose asthma remains uncontrolled on standard medication.

## Methods

**Figure 1:**
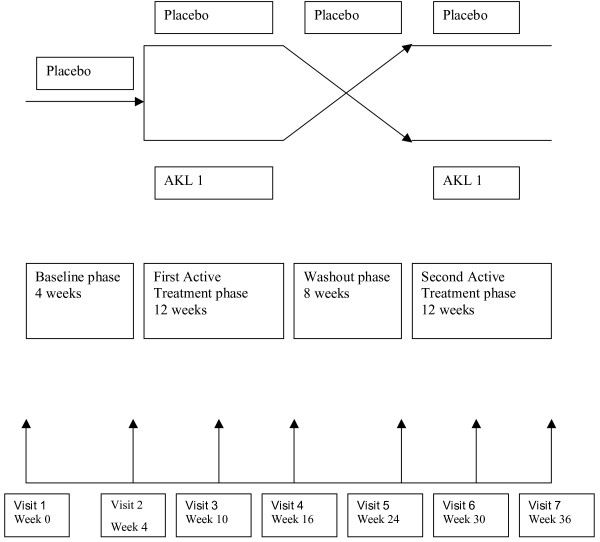
Study Schematic.

This was a randomised, double-blind, placebo controlled, cross-over study assessing the efficacy of orally administered AKL1 capsules in addition to the normal prescribed medication for the treatment of asthma, which continued for the duration of the study. Consenting adults with sub-optimally controlled asthma despite current therapy (which included regular ICS treatment) were recruited. The study consisted of four periods; a four week single-blind baseline period, during which the subject continued normal treatment and in addition took a placebo capsule, a twelve-week double-blind treatment period during which patients received either AKL1 or indistinguishable placebo in addition to normal treatment, a eight week single-blind washout period during which a placebo was taken and a further twelve-week double-blind treatment period during which patients crossed over from active treatment to placebo or vice-versa. Subjects were randomly assigned to receive either AKL1 or placebo in the first and second active phase in a double-blinded manner (Figure 1).

### Subjects

Consenting adult patients aged between 18 and 75 years with persistent asthma (defined as per GINA criteria [17]) and currently receiving inhaled corticosteroid medication at a total daily dose of 400–2000 mcg beclomethasone diproprionate or equivalent were recruited by advertisements in local Doctor's surgeries and in the local newspaper. All patients had a documented positive bronchodilator reversibility test with ≥ 15% improvement in FEV_1 _from 15 to 30 minutes after inhalation of at least 200 μg of salbutamol (beta-2-adrenergic agonist administration) or documented PEF variability of 20% as described in BTs guidelines [18]. Exclusion criteria included documented COPD and unstable asthma defined the requirement for oral corticosteroids and/or admission to hospital for asthma (including treatment in an emergency room) in the prior three months.

Subjects completed a daily diary of bronchodilator use and measured Peak Expiratory Flow (PEF) rate each morning (pre-bronchodilator) and evening, utilising a PiCo electronic PEF meter [19] with data transmitted real-time via a mobile phone link using the eSAN electronic peak flow meter and diary system [20]. (Due to technical problems with cell phone coverage and data transmission problems, 8 patients elected to use paper diary cards and a mini-Wright's peak flow meter (Clement Clarke International Ltd) to collect identical information). At study visits (Table 1), spirometry was measured (Microlab), questionnaires were administered (Asthma Control Questionnaire [21], Asthma Quality of Life Questionnaire [22], Leicester Cough Questionnaire) [23] and blood was drawn for haematological and biochemical profiles, and data was collected on adverse events, asthma exacerbations and non-study medication use.

**Table 1:** Schedule of events

**Visit No**	**1**	**2**	**3**	**4**	**5**	**6**	**7**
	**Baseline**	**Randomisation: Start treatment phase 1**	**Mid-Treatment phase 1**	**End of Treatment phase-Washout**	**Start Treatment phase 2**	**Mid-Treatment phase 2**	**End treatment, Final visit**

**Week No.**	0	4	10	16	24	30	36
**Consent**	X						
**Inclusion/exclusion criteria**	X	X					
**Physical exam**	X						X
**Vital signs**	X	X	X	X	X	X	X
**Blood drawn**	X			X			X
**Pregnancy test**	X	X			X		
**Bronchodilator reversibility**	X						
**Spirometry**	X	X	X	X	X	X	X
**Asthma exacerbations**	X	X	X	X	X	X	X
**AQLQ**	X	X	X	X	X	X	X
**ACQ**	X	X	X	X	X	X	X
**LCQ**	X	X	X	X	X	X	X
**Bronchodilator use**	X	X	X	X	X	X	X
**Dispense study medication**	X	X	X	X	X	X	
**Randomisation**		X					

In order to be randomised at visit 2, subjects had to use ≥ 2 puff per day and ≤ 12 puffs per day of rescue medication (salbutamol) on average during the 7 days immediately prior to the visit, and to have experienced no exacerbation during the lead-in period requiring additional therapy.

### Outcome measures

The primary outcome measure was the effect of AKL1 on lung function (Peak Expiratory Flow Rate, PEF), comparing for each patient the change in mean morning pre-bronchodilator PEF between the final week of the run-in or wash-out period and the final week of active treatment minus the change in PEF over the same parameters for placebo treatment.

Secondary endpoints were comparisons of the changes in individual patients over the active treatment and placebo phases of the following parameters:

• Forced expiratory volume in 1 second (FEV_1_) measured at study visits

• Asthma related health status (Juniper Asthma Quality of Life Questionnaire scores, AQLQ)

• Asthma control (Juniper Asthma Control Questionnaire scores, ACQ)

• Cough related health status (Leicester cough questionnaire scores, LCQ)

• Asthma exacerbations (defined as the need for the use of short burst oral corticosteroid medication)

• Average short acting beta agonist (SABA) rescue medication use.

In addition, adverse events were recorded and the following parameters were monitored:

• Blood pressure

• Full Blood Count

• Urea and Electrolytes

• Liver Function Tests

### Sample size

Assuming that the within-patient standard deviation of peak flow is 40 Liters/min, a study population of 30 patients will detect a treatment difference in PEF of 30 L/min at a two-sided 5% significance level, with a power of 80%. To allowing for an estimated drop-out rate of up to 25% we therefore aimed to enroll 40 patients

### Ethical approval

The study received ethical approval from the Grampian Local Research Ethical Committee

### Statistical analysis

The data were entered into an Access databases and analyzed using SPSS version 14. Paired data were analyzed with the paired Student's T test. Descriptive demographic statistics are presented as mean and standard deviation (SD) for normally distributed data and median and interquartlie range (IQR) for skewed data. Changes in outcome measures over the treatment phases are presented as means and corresponding 95% confidence intervals (95%CIs). A p value of less than 0.05 was taken to signify statistical significance.

## Results

### Patient demography

Sixty subjects were assessed at the screening visit and 43 subjects met the entry criteria and were randomised. Eleven patients failed to complete the study; 7 withdrew consent and 4 became uncontactable during the study. Thirty two patients completed the full study protocol and provided data for analysis.

The median (IQR) age of the per-protocol population was 40.5 (33.7–55.2) yrs, and 25 subjects were female. The mean (SD) morning pre-bronchodilator PEF during baseline period was 386 (106) L/min, the median (IQR) FEV1 2.65 (1.93–2.95)L, median (IQR) FEV1 % predicted was 87.5 (74.2–108.7)%. The median (IQR) daily inhaled corticosteroid dose was 800 (400–1700) mcg/day. 15 subjects received long acting beta agonists, 3 received leukotriene receptor antagonists and 2 received theophyllines in addition to inhaled corticosteroids and as required short acting beta agonists. 15 subjects reported allergies, and 7 a history of allergic rhinitis or hay fever. The subjects' usual medical attendants were requested not to alter maintenance asthma treatment during the study, and no changes in asthma treatment other than short burst changes relating to asthma exacerbations (see below) occurred.

The mean (SD) AQLQ score at baseline were 4.76 (0.92), ACQ score 1.83 (0.75), the median (IQR) LCQ score 5.66 (4.42–6.25), indicating sub-optimal asthma control and moderately impaired asthma-related and cough-related health status at baseline.

No significant differences in baseline parameters were observed between the group randomised to receive active treatment followed by placebo and the group randomised to receive placebo followed by active treatment in age, sex, current smoking status, inhaled corticosteroid dose or percentage predicted FEV1 (table 2).

**Table 2:** Baseine characteristics (age, sex, percentage predicted Forced expiratory rate in the first second (FEV1), Inhaled Cosirosteroid (ICS) dose in mcg/dat beclomethasone equivalent, ciuurent smoking status in groups randomsied to AKL followed by placebo or to placebo followed by AKL, with p values for differences between groups.

	**age (median, IQR)**	**Female sex (n)**	**FEV1 % predicted (median, IQR)**	**ICS dose, mcg/day (median, IQR)**	**Current smoker (n)**
**AKL1 – Placebo (n = 16)**	41.00, 33.50 – 55.75	13	90.00, 79.75 – 115.50	1000, 400 – 2000	1
**Placebo – AKL1 (n = 16)**	35.50, 29.50 – 55.50	12	85.50, 56.50 – 110.00	800, 400 – 1150	1
**P value**	0.67	0.67	0.25	0.51	1.00

### Lung function changes

#### 1. PEF

**Figure 2:**
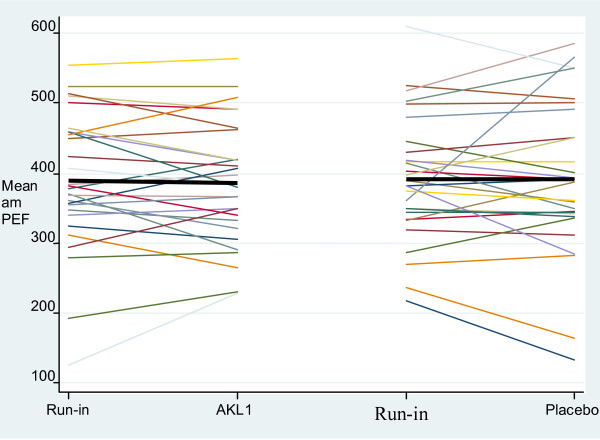
Change in individual subject mean morning PEF over the active treatment and placebo treatment phases (the colored lines represent individual patients and the black bar the grouped mean change).

The individual patient changes in mean morning pre-bronchodilator PER from the week preceding treatment phase (i.e. the last week of run-in or of wash-out) to the last week of active treatment phase are shown in Figure 2. The mean difference (95% CI) in the change in mean morning PEF for individual subjects between active treatment and placebo (effect of AKL1 – effect of placebo) was -4.1 (-35.0 to 26.9) l/min (p = 0.8), indicating no significant change in PEF as a result of AKL1 treatment.

#### 2. FEV1

The mean difference (95% CI) in change in FEV1 for individual subjects between active treatment and placebo (effect of AKL1 – effect of placebo) was 0.01 (-0.12 to 0.14) litres (p = 0.9), again indicating that AKL1 resulted in no significant change in lung function.

### Questionnaire based patient-centred outcome changes (Table 3)

**Table 3:** Mean Changes in Asthma Control Questionnaire (ACQ), Asthma Quality of Life Questionnaire (AQLQ) and Leicester Cough Questionnaire (LCQ) scores associated with AKL1 treatment (AKL1 – Placebo).

**Questionnaire**	Mean difference (95% CI)*	P-value**
**ACQ**	-0.35 (-0.78 to 0.07	0.10
**AQLQ**	0.43 (-0.08 to 0.93)	0.09
**LCQ**	0.49 (-0.18 to 1.16)	0.14

* Negative score on ACQ indicate improved asthma control. Positive scores on AQLQ and LCQ indicate improved health status

**Students paired T Test

#### 1. Asthma heath status: AQLQ

**Figure 3:**
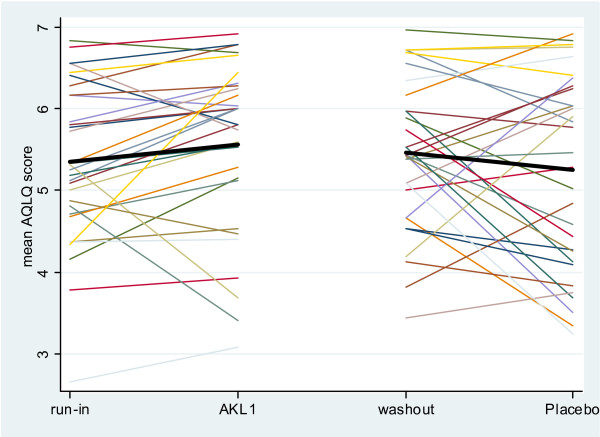
Change in individual subject Asthma Quality of Life Questionnaire (AQLQ) score over the active treatment and placebo treatment phases (the colored lines represent individual patients and the black bar the grouped mean change, higher reading equates to improved health status).

The individual subject changes in AQLQ scores over the active treatment and placebo phases are shown in Figure 3. The difference in change in individual subject mean AQLQ score (effect of AKL1 – effect of placebo) showed a trend to improvement with AKL1 treatment of 0.42 (-0.08 to 0.93), p = 0.09.

With an individual patient change in AQLQ score of 0.5 signifying a clinically relevant change in health status [24], 42% of subject improved on AKL1, 29% of subjects improved on placebo and 29% had similar health status changes on AKL and placebo.

#### 2. Asthma control: ACQ

**Figure 4:**
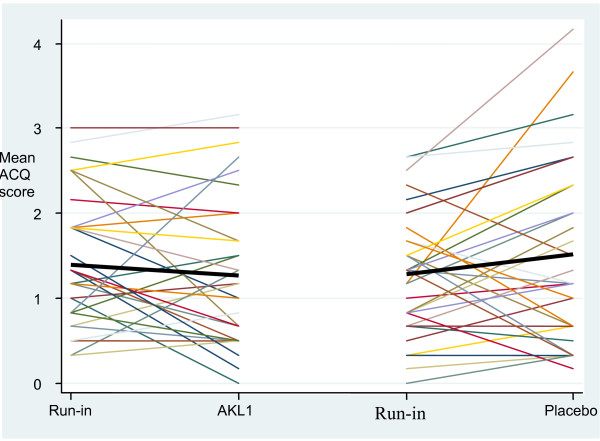
Change in individual subject Asthma Control Questionnaire (ACQ) score over the active treatment and placebo treatment phases (the colored lines represent individual patients and the black bar the grouped mean change, a lower score equates to improved asthma control).

The changes in individual subject ACQ scores over the active treatment and placebo phases are shown in Figure 4. The difference in change in individual subject mean ACQ score (effect of AKL1 – effect of placebo) showed a trend to improvement with AKL treatment of -0.35 (95% CI of the difference -0.78 to 0.07), p = 0.10.

With an individual patient change in ACQ score of 0.5 signifying a clinically relevant change in health status [24], 50% of subject improved on AKL1, 22% of subjects improved on placebo and 28% had similar health status changes on AKL and placebo.

#### 3. Cough related health status (LCQ score)

The difference in change in individual subject LCQ score (effect of AKL1 – effect of placebo) showed a trend to improvement with AKL1 treatment of 0.49 (-0.18 to 1.16), p = 0.15.

## Adverse outcomes

### Asthma exacerbations (Table 4)

**Table 4:** Number of exacerbations by occurring during active treatment phases

		**Number of exacerbations while on Placebo**			Total
			
		**0**	**1**	**2**	
**Number of exacerbations while on AKL1**	**0**	25	2	1	28
	**1**	0	1	2	3
	**2**	1	0	0	1

Total		26	3	3	32

Only seven of the 32 patients in this study reported the occurrence of exacerbations during the active treatment phases. A total of 9 exacerbations occurred whilst taking placebo (3 patients having a single exacerbation and 3 patients having 2 exacerbations) and a total of 5 with AKL1 (3 patients having single exacerbation and one patient having 2 exacerbations.

### Other adverse events

Sixty non-asthma related adverse events were recorded during the study, most related to inter-current infections or minor illnesses; none were deemed to be treatment related. 22 events occurred while on AKL treatment and 38 whilst on placebo (data available on request)

No significant changes in weight, pulse rate, blood pressure, hematological or biochemical parameters were found across the study period (data not presented, available on request).

## Discussion

This randomised controlled double blinded cross-over trial compared the effects of 12 weeks of treatment with the encapsulated botanical mixture AKL1 on lung function, asthma control, asthma and cough related health status and on treatment related adverse outcomes with that of 12 weeks of indistinguishable placebo capsules as add-on treatment for patients having sub-optimally controlled asthma on standard therapy at study entry.

The primary outcome of the study was lung function. No significant or potentially relevant changes in lung function were found between active and placebo treatment. It is therefore very unlikely that AKL1 has an effect on lung function in asthma. No statistically significant changes secondary outcomes were found, but consistent trends were however seen towards improvements in patient centered asthma outcomes, including symptomatic asthma control (ACQ scores), asthma related health status (AQLQ scores) and cough related health status (LCQ scores). Although none of these outcome measures individually reached statistical significance, similar trends were seen towards improvement with AKL1 treatment over placebo for each. It is possible that the study was insufficiently powered to demonstrate a clinically and statistically relevant effect for these outcome measures. The magnitude of the effect on asthma control and asthma related quality of life appeared to have not been inconsiderable; comparing the proportions of subjects showing a clinically relevant change in asthma control in association with AKL1 treatment, 50% improved while 22% deteriorated, and for asthma-related health status, 42% improved while 29% deteriorated. No safety concerns emerged over AKL1 during the trial, with a numerical decrease in asthma exacerbations and an adverse event rate similar to placebo. The consistent trends to improvement in patient centered outcomes warrant further consideration and investigation

These data point to a possible dissociation between lung function and patient centered outcomes in this study. How are we to understand this dissociation? One possible explanation is that as patients were recruited from the community with mild to moderate asthma, lung function impairment may have been too mild to demonstrate an improvement on treatment. However, the lack of even a trend for lung function tests effects makes it unlikely that this fully explains this dissociation. It is well recognized however that asthma is a complex and heterogeneous disease that affects patients in a variety of ways, and that no single outcome measure can encompass the complexity of asthma [25]. Poor correlations have been reported in previous studies between lung function and patient centred outcomes such as asthma symptoms [26], and composite outcomes encompassing a variety of measures including patient centered parameters are increasingly used [27]. If AKL1 is indeed having an effect on asthma, it may be that it is affecting manifestations of the disease independent of bronchspastic phenomena, and may result in improvements in symptoms and hence the impact of the disease on the individual independently of any effects on bronchoconstriction. Further research is needed to investigate this possibility.

The strengths of this study were that it recruited asthmatics from the community (where most asthma is now treated) with a typical demographic profile, and it used a rigorous scientific methodology to control for the many potential confounding factors that may bias the results of investigations of CAM therapies. A weakness is the size of the study; as only 32 patients completed the study, based on a sample size calculation for a change in PEF of 30 l/min, there were insufficient numbers to provide a statistically significant result in the secondary patient centered outcome measures or in exacerbations. A further, larger study is now required to investigate the effects of AKL1 on symptom control, exacerbations and health status in asthma. In addition, the study only investigated the use of AKL1 as an add-on therapy, and no information can be inferred on the use of this agent as monotherapy or as a steroid-sparing agent.

This study recruited patient treated in the community for mild to moderate asthma whose symptoms were not fully controlled on standard therapy; surveys have shown that many asthmatic patients do indeed have inadequate control [1,28,29], and that many patients are willing to put up with inadequate control in order to limit their perceived dependence on conventional preventative pharmacotherapy [1,29,30]. Herbal treatments are often perceived as being more 'natural' and so more acceptable than standard pharmacological agents, and so the demonstration of effectiveness for a botanical based treatment would have the potential to offer a useful adjuvant treatment to many asthmatics. The side-effect and adverse event profile of AKL was found to be excellent in this study.

## Conclusion

This study used a cross-over randomised controlled methodology to investigate whether AKL1, an encapsulated botanical mixture, was more effective than indistinguishable placebo as an add-on treatment for patients whose asthma remained uncontrolled on standard pharmacotherapy. No effect was observed on lung function, and no statistically significant effects were seen in patient centered outcomes, although consistent trends were observed to improvements in asthma symptomatic control, asthma health status and cough health status. The tolerability profile of AKL1 was excellent. Further research is required to investigate the possibility that this treatment may be an effective add-on treatment for asthma.

## Abbreviations

ICS: Inhaled Corticosteroids

CAM: Complementary and Alternative Medicine

GINA: Global Initiative in Asthma

FEV1: Forced Expiratory Volume in first second

PEF: Peak Expiratory Flow Rate

COPD: Chronic Obstructive Pulmonary Disease

ACQ: Asthma Control Questionnaire

AQLQ: Asthma Quality of Life Questionnaire

LCQ: Leicester Cough Questionnaire

SABA: Short Acting Beta Agonist

SD: Standard Deviation

IQR: Interquartile Range

## Competing interests

The author(s) declare that they have no competing interests.

## Authors' contributions

MT devised the protocol, supervised the study and drafted the manuscript.

JS and NS performed the study, entered the data into the study database and assisted in the production of the manuscript.

SF and AL analysed the data and contributed to the manuscript.

All authors read and approved the final manuscript.

## Pre-publication history

The pre-publication history for this paper can be accessed here:


